# Overexpression of FGFR1 in AgRP neurons protects against obesity and improves glucose homeostasis

**DOI:** 10.3389/fendo.2026.1905737

**Published:** 2026-08-31

**Authors:** Daniel Shookster, Ahjanelle Martin, Madison Gunn, Shea O’Connell, Hu Huang

**Affiliations:** 1 East Carolina Diabetes and Obesity Institute, East Carolina University, Greenville, NC, United States; 2 Department of Kinesiology, East Carolina University, Greenville, NC, United States; 3 Human Performance Laboratory, College of Human Performance and Health, East Carolina University, Greenville, NC, United States; 4 Department of Physiology, East Carolina University, Greenville, NC, United States

**Keywords:** AgRP neuron, FGFR1 expression, glucose homeostasis, metabolism, obesity

## Abstract

**Background:**

The rising prevalence of metabolic disorders highlights the need to identify targetable pathways. Although hypothalamic AgRP neuron signaling influences energy balance, the beneficial role of fibroblast growth factor receptor 1 (FGFR1) within these neurons remains incompletely understood.

**Methods:**

We employed an AAV-mediated CRISPR/Cas9 activation system to achieve AgRP neuron-specific overexpression of FGFR1 in adult mice. Metabolic phenotypes were assessed during high-fat diet (HFD) feeding, and mechanistic insights were gained using GT1–7 hypothalamic cells.

**Results:**

FGFR1 overexpression in AgRP neurons attenuated high-fat diet (HFD)-induced weight gain and improved glucose tolerance, effects that were associated with reduced energy intake. Mechanistically, FGFR1 overexpression correlated with increased FOXO1 phosphorylation and reduced AgRP mRNA expression, suggesting a potential FGFR1-FOXO1 regulatory axis.

**Conclusion:**

These findings identify a contributory role for AgRP neuronal FGFR1 signaling in modulating metabolic outcomes under HFD conditions and suggest that this pathway may represent a potential avenue for further investigation in the context of obesity and diabetes.

## Introduction

The escalating global prevalence of obesity and type 2 diabetes underscores the critical need to elucidate the molecular mechanisms governing metabolic homeostasis. The central nervous system, particularly the hypothalamus, serves as the master regulator of energy balance, integrating peripheral hormonal and nutrient signals to coordinate feeding behavior, energy expenditure, and glucose metabolism (1–3). Within the arcuate nucleus (ARC) of the hypothalamus, distinct populations of first-order neurons serve as sensors for circulating factors, including leptin, insulin, ghrelin, and glucose (4–6). Among these, the orexigenic agouti-related peptide (AgRP) neurons are particularly crucial; their activation is both necessary and sufficient to drive voracious feeding, while their inhibition promotes satiety and weight loss (7, 8). In metabolic disorders, the exquisite sensitivity of these neurons to hormonal cues is often blunted—a state known as leptin and insulin resistance—leading to a failure in homeostatic feedback and the perpetuation of positive energy balance (9, 10). Therefore, identifying signaling pathways that can modulate AgRP neuron activity and restore their sensitivity presents a promising therapeutic strategy.

The fibroblast growth factor (FGF) family, especially through fibroblast growth factor receptor 1 (FGFR1), has recently emerged as a key player in metabolic regulation. FGFR1, a widely expressed receptor tyrosine kinase, is notably abundant within the central nervous system, including the hypothalamus (11, 12). A growing body of evidence implicates central FGF signaling in the control of energy and glucose homeostasis. For instance, central administration of FGF19 or FGF21 can potently reduce body weight and improve glycemia (13–16). Importantly, some of these metabolic effects appear to be mediated specifically through AgRP neurons (15). Recent studies have shown that FGF ligands, upon central administration, require FGFR1 expression in AgRP neurons to exert their anti-diabetic effects, suggesting a direct and crucial role for this receptor in a defined neuronal subset (17–20). However, much of the existing literature has relied on the administration of exogenous ligands, which may activate multiple receptor isoforms or indirect pathways. This approach leaves a fundamental question unanswered: What is the neuron-specific contribution of FGFR1 signaling within AgRP neurons? The specific contribution of the receptor itself, independent of exogenous ligand administration, remains poorly defined.

Notably, FGFR1 expression in the hypothalamus is dynamically regulated by nutritional status. Studies in rodents show that prolonged high-fat diet (HFD) feeding significantly suppresses FGFR1 mRNA levels, implying that diminished FGFR1 signaling may be a pathological feature of obesity (14). Supporting this, we recently found that specific deletion of FGFR1 in AgRP neurons leads to an obese and diabetic phenotype, indicating that FGFR1 downregulation contributes to metabolic dysfunction (21, 22). Based on these findings, we hypothesize that overexpressing FGFR1 specifically in AgRP neurons could protect against diet-induced metabolic impairments. To test this hypothesis directly and circumvent the limitations of ligand-based studies, we employed a targeted genetic approach. We used an adeno-associated virus (AAV)-mediated CRISPR activation (CRISPRa) system to achieve selective overexpression of FGFR1 in AgRP neurons of adult mice. This innovative technique utilizes a catalytically dead Cas9 (dCas9) fused to transcriptional activators, allowing for precise upregulation of the endogenous FGFR1 gene without altering its genomic sequence (23–25). Following viral delivery, mice were subjected to an HFD challenge to model human metabolic disease. We then conducted comprehensive metabolic phenotyping, assessing longitudinal changes in body weight, adiposity, food intake, glucose tolerance, insulin sensitivity, and energy expenditure. Our findings provide direct genetic evidence that enhancing FGFR1 signaling specifically within AgRP neurons is sufficient to confer protection against diet-induced obesity and glucose intolerance, thereby suggesting that FGFR1 serves as a contributory regulator of metabolic physiology in this key neuronal population.

## Methods

### Experimental mice

All experimental protocols were approved by the Institutional Animal Care and Use Committees of East Carolina University, and mice were cared for following the National Institutes of Health Guide for the Care and Use of Laboratory Animals. AgRP-IRES-Cre mice (JAX 012899) were housed at 20–22 °C with a 12-h light-dark cycle (7:30 AM light/7:30 PM dark). Mice were fed a high-fat diet (Research diet D12331) with ad libitum access to water from 6 weeks of age throughout the study (22 weeks of age).

### Stereotaxic injections and viral constructs

Six-week-old mice were anesthetized with 2-4% isoflurane (delivered in 100% O_2_ at a flow rate of 1 L/min via nose cone) and then placed into a stereotaxic device (KOPF model 922), after which eye ointment was applied and a small incision was made to expose the skull. A cordless micro-drill (Stoelting, Wood Dale, IL) was then used to drill a small hole in the skull to allow for needle passage into the brain. Bilateral injections of viral constructs (250 nl/side) were targeted to the ARC according to coordinates from bregma, anterior-posterior (AP): -1.40 mm, dorsal-ventral (DV): -5.85 mm, left-right (LR): ± 0.3 mm at a rate of 30 nl/min using a 0.5 µl Hamilton syringe (Neuros model 7000.5 KH). Following injections, the needle was left in position for an additional 10 min to permit virus diffusion prior to withdrawing the needle.

**Figure 1:**
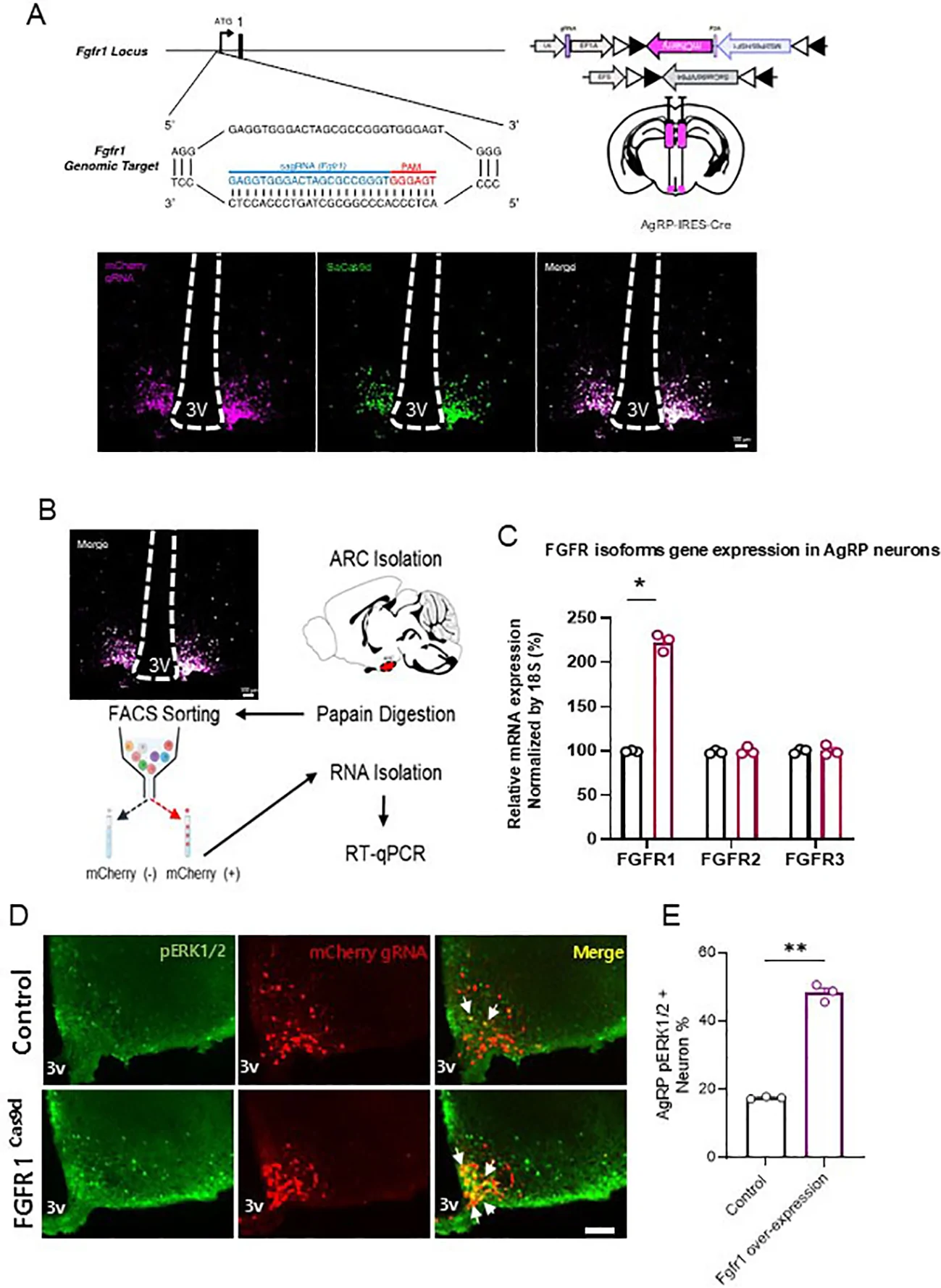
Validation of a CRISPR/dCas9 system for cell-type-specific FGFR1 overexpression. **(A)** Schematic diagram of viral constructs bilaterally injected into the ARC of AgRP-IRES-Cre mice, and Co-immunostaining of mCherry and SaCas9d expression in AgRP neurons. **(B)** Schematic Diagram of experimental workflow for FACS sorting of AgRP neurons based on mCherry expression. **(C)** FGFR1, 2, and 3 mRNA expression following CRISPR-Cas9d-mediated overexpression of FGFR1 in AgRP neurons, n=3 per group (Welch’s t test, Two-tailed t=61.51, df=2.370, p<0.0001). **(D)** Representative immunofluorescent images of AgRP neurons (Red) colocalization with pERK1/2 (green). **(E)** Quantification of the percentage of pERK1/2 colocalization in AgRP neurons, n=3 per group (Welch’s t test, two-tailed, t=20.87, df=2.077 p=0.0019). All data are presented as mean ± s.e.m *P < 0.05, **P < 0.01, ***P < 0.001.

We designed two AAV vectors, one carrying a scramble/control gRNA, and one targeting FGFR1 to ensure the observed phenotype did not arise from a component of the CRISPR activation system (**Figure 1A**). Mice were injected with 1:1 mix of AAV8-sagRNAMS2/P65/HSF1(FGFR1)-FLEX-mCherry (7.86 x 1013 GC/ml) or AAV8-sagRNAMS2/P65/HSF1(Scramble)-FLEX-mCherry (8.25 x 1013 GC/ml) and AAV8-FLEX-dSaCas9/VP64 (4.89 x 1013 GC/ml). We tested the gRNA sequences using hypothalamic GT1–7 cells, observing a three-fold increase of FGFR1 expression in cells transfected with FGFR1Cas9d gRNA compared to control/scramble gRNA-transfected cells. All viral constructs were sourced from VectorBuilder Inc. (Chicago, IL, USA) and stored at -80 °C until use. Based on the manufacturer’s description and online CRISPR tool (https://www.zlab.bio/resources), RT-qPCR was performed using the primer sequence GAGGTGGGACTAGCGCCGGGTGGGAGT, and a scramble sgRNA (GCGCGATAGCGTCTCCGCT) was used as a control. To ensure data quality and interpretability, only mice with viral expression confirmed to be restricted to AgRP neurons (by IHC mCherry expression within AgRP) and with verified FGFR1 overexpression (by qPCR) were included in the final analysis. Mice with off-target expression, incomplete targeting, or insufficient FGFR1 modulation were excluded prior to phenotypic assessment. Approximately 50% of injected animals (~6 mice per group) were excluded based on these criteria, yielding a final sample size of n = 6–8 mice per group with confirmed targeting. For postoperative care, mice were injected intraperitoneally with meloxicam (0.5 mg/kg BW) for three consecutive days, with their body weight and health conditions being closely monitored during recovery.

### Food intake, body weight, energy expenditure, and body composition measurements

Following AAV injections, food intake and body weight were measured daily during the two-week recovery period and weekly thereafter. Body weight and food intake were measured between 8–9 AM using an OHAUS scale (VALOR™ 2000, OHAUS, Parsippany, NJ, USA). Cages were inspected for food pellets and partially eaten food bits during measurements to facilitate accurate measurements of food intake. For energy expenditure measurements, 12-week-old mice were housed individually in TSE PhenoMaster metabolic cages (TSE PhenoMaster, Berlin, Germany). Mice became accustomed to the metabolic cages for three days before recording, with the energy expenditure data being collected during days 4–6 to be used for analysis. Energy expenditure data were normalized by lean mass and analyzed with the online CalR tool (26, 27). At the end of the TSE metabolic cages experiment, body composition was determined using Echo MRI (Echo MRI 3-in-1 composition analyzer, Echo Medical Systems, Houston, TX).

### Glucose and insulin tolerance tests

At 14 weeks of age (8 weeks post-AAV), mice underwent glucose tolerance tests by intraperitoneal injections using a 20% glucose solution (1.0 g/kg BW) after fasting overnight. Insulin tolerance tests were performed at 16 weeks of age (10 weeks post-AAV) by intraperitoneal injection of 0.6 U/kg insulin after a 4-hour fast. Blood glucose levels were measured from a drop of tail blood at 0, 15, 30, 60, 90, and 120-minute time intervals during the tests using a handheld glucometer (ReliOn Prime Blood Glucose Monitoring System; ARKRAY Inc., Kyoto, Japan).

### Quantitative real-time PCR

RNA was isolated from tissues or cells using the RNeasy Micro Kit (Qiagen, 74004) according to the manufacturer’s protocol. Following isolation, RNA was reverse transcribed into cDNA using the Applied Biosystems high-capacity cDNA reverse transcription kit (Thermo Fisher, 4368814) according to the manufacturer’s protocol. RT-qPCR was then performed in triplicate using the Applied Biosystems ViiA 7 system, with mRNA expression normalized to the mouse 18s ribosomal RNA housekeeping gene and expression fold change calculated using the 2−ΔΔCt method. Primer sequences are listed in **Supplementary Table 1**.

### Western blot

Western blot procedures were performed as previously described (6). In brief, following protein extraction according to standard protocol, the Pierce BCA Protein Assay Kit was used to assess protein concentration. Electrophoresis was conducted on a 4–20% Criterion™ Tris-HCl Protein Gel loaded, then transferred to a nitrocellulose membrane and blocked with 5% non-fat milk TBS-T for 2 hours. Following the blocking period, primary antibodies pFOXO1ser256, total FOXO1, or FGFR1 (Cell Signaling Technology) were applied at 1:1000, after which the membranes were incubated at 4°C overnight. The following day, secondary antibodies IgG-HRP 1:1000 (Santa Cruz Biotechnology) were applied, followed by incubation at room temperature for 2 hours. Membranes were washed with TBS-T between each procedure, and immunoreactive bands were visualized using ECL methods and imaged with Bio-Rad ChemiDoc.

### Cell culture and transfection

Hypothalamic GT1–7 cells were cultured at 37 °C, 5% CO2 in high-glucose (4.5 mg/dL) DMEM, 10% FBS, and 1% penicillin-streptomycin with adenovirus Cre to permit the recombination of Cre-dependent constructs. Cells were then transfected with Lipofectamine 3000 (ThermoFisher Scientific) according to the manufacturer’s protocol using DNA plasmids of pAAV-sagRNAMS2/P65/HSF1(FGFR1)-FLEX-mCherry or pAAV-sagRNAMS2/P65/HSF1(Scramble)-FLEX-mCherry and pAAV-FLEX-dSaCas9/VP64. Cells were incubated for 72 hours following transfection, after which cells were harvested, and protein was extracted for electrophoresis.

### Immunohistochemistry

After induction of deep anesthesia with 2-4% isoflurane inhalation (delivered at 1 L/min O_2_ via nose cone), and confirmation of a surgical plane of anesthesia by toe pinch, mice were intracardially perfused with PBS followed by 10% formalin (Epredia™ Formal-Fixx™), after which brains were incubated at 4 °C in 10% formalin for 48 h before being stored in 30% sucrose until sectioning. Brains were sectioned into 30 μm coronal sections using a freezing microtome (VT1000 S; Leica, Wetzlar, Germany). After sectioning, brain slices were washed in PBS (6 × 10 min), then blocked for 1 h in PBST & normal donkey serum (Jackson ImmunoResearch Inc., West Grove, PA, USA), then incubated overnight with primary antibodies. The following day, sections were washed in PBS (6 × 10 min) and then incubated with Alexa-fluorophore secondary antibodies (Abcam, Waltham Boston, MA, USA) for 1 hour. Sections were then mounted on SuperFrost slides (Fisher Scientific) with VECTASHIELD^®^ Antifade Mounting Medium with DAPI H-1200-10 (Vector Laboratories Inc., Burlingame, CA, USA). Images were taken on a Keyence BZ-X810 microscope (Keyence, Itasca, IL, USA), using 10x and 20x objective lenses and analyzed with Fiji software.

### Fluorescence activated cell sorting

After euthanasia via deep anesthesia with 5% isoflurane inhalation (delivered via vaporizer in an induction chamber until respiration ceased) followed by cervical dislocation, the brains were rapidly removed. The ARC was dissected from the hypothalamus and then enzymatically digested using Papain dissociation (Worthington Papain), as described previously (28). Following dissociation, Dapi was added to the suspension to assist cell sorting to ensure cell health. Afterwards, cells were sorted using a BD FACS Aria Fusion device (BD Bioscience). Only cells that were mCherry-positive and DAPI-negative were used to extract RNA for quantitative real-time PCR. Cell sorting was conducted using measurements of mCherry and Dapi fluorescence (mCherry: excitation 561 nm, detection: bandpass 610 ± 20 nm, Dapi: excitation 405 nm, detection: bandpass 450 ± 50 nm).

### Statistical analyses

Data are presented as mean ± SEM, and relevant statistical test information and results are presented in the figure legends. Analyses were made using GraphPad Prism statistic software versions 8 & 9 (GraphPad Software, La Jolla, CA) with a P value <0.05 considered statistically significant.

## Results

### A Cre-dependent viral approach for targeted FGFR1 overexpression in adult AgRP neurons

To delineate the metabolic functions of FGFR1 specifically within arcuate nucleus (ARC) AgRP neurons, we implemented a targeted gain-of-function strategy in adult mice. This approach utilized an Adeno-Associated Virus (AAV) delivering a Cre-dependent CRISPR-Cas9 activation (CRISPRa) system, specifically the catalytically dead *Staphylococcus aureus* Cas9 (dSaCas9), in conjunction with AgRP-Cre transgenic mice (**Figure 1A**). This methodology enables selective manipulation of FGFR1 expression in adulthood, thereby circumventing potential developmental compensatory mechanisms and the off-target effects associated with chronic pharmacological inhibition, as underscored by studies demonstrating the high plasticity and compensatory capacity of energy balance circuits (29–31).

To validate the specificity of the viral injection, we confirmed that co-expression of the fluorescent marker mCherry and dSaCas9 was restricted to the ARC and absent from the adjacent ventromedial hypothalamus, which lacks Cre recombinase expression in this model (**Figure 1A**). Control animals received a co-injection of dSaCas9 and a non-targeting (scramble) gRNA to account for potential non-specific effects of the CRISPRa machinery itself.

The efficacy of CRISPR/dSaCas9-mediated FGFR1 overexpression was assessed *in vivo*. mCherry-positive AgRP neurons were isolated by fluorescence-activated cell sorting (FACS), followed by analysis of FGFR isoform mRNA levels via RT–qPCR (**Figures 1B, C**). This analysis revealed a significant increase in FGFR1 mRNA expression in mice transduced with the FGFR1-specific sgRNA compared to scramble gRNA-injected controls. In contrast, mRNA levels of other isoforms, including FGFR2 and FGFR3, were unaltered. Furthermore, all three FGFR isoforms remained intact in mCherry-negative non-AgRP neurons, in which AgRP mRNA was undetectable (**Supplementary Figure 1**). Additionally, phosphorylation of ERK1/2 (pERK1/2)—a downstream marker of FGFR1 activation was also significantly elevated relative to controls (**Figures 1D, E**), collectively supporting effective, AgRP neuron-specific overexpression of FGFR1 using CRISPR/Cas9a.

### Overexpression of FGFR1 in AgRP neurons mildly protects against obesity and improves glucose metabolism during HFD feeding

**Figure 2:**
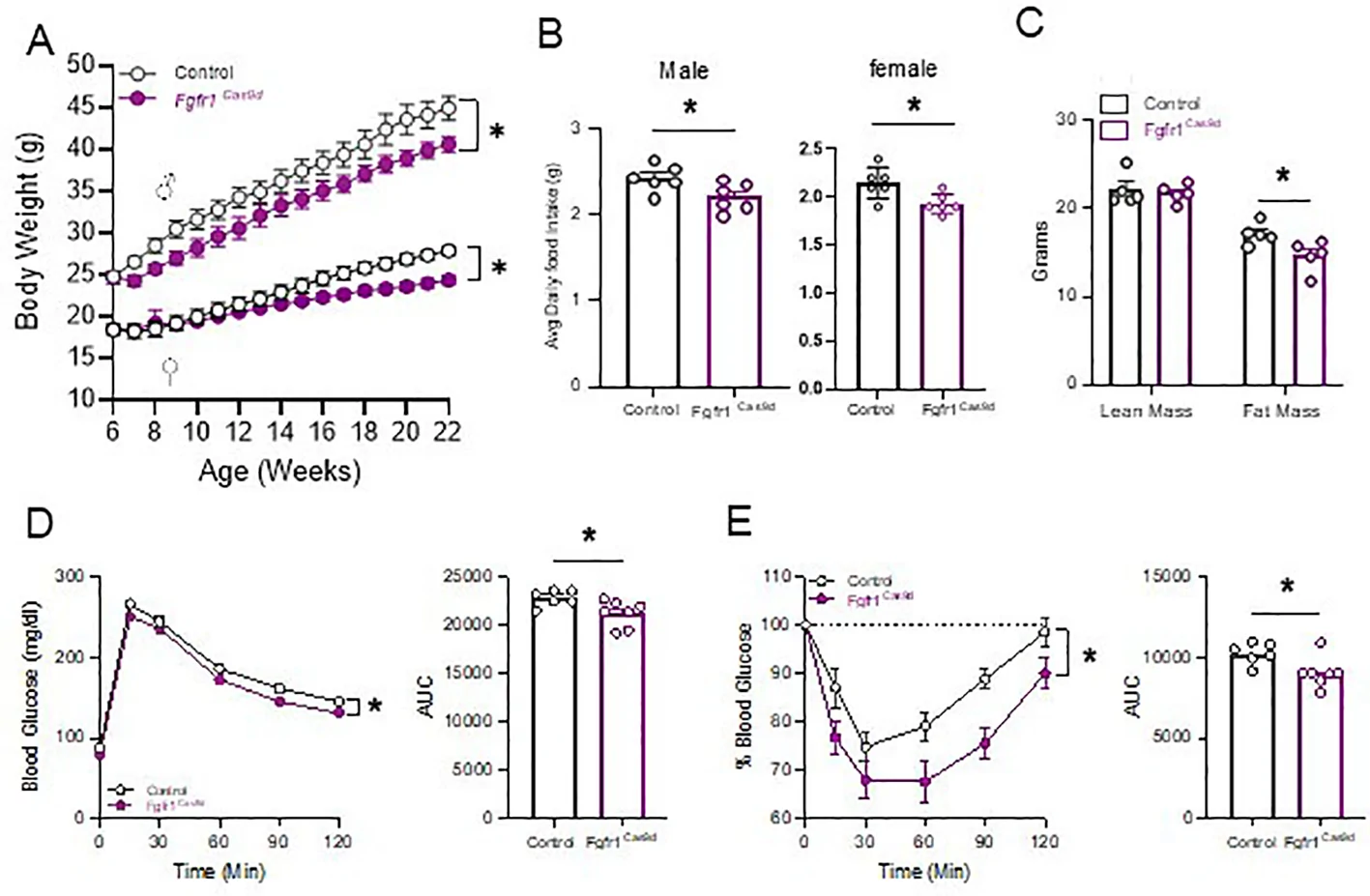
Overexpression of FGFR1 in AgRP neurons mildly protects against obesity and improves glucose metabolism during HFD feeding. **(A)** Both male mice bodyweight curve (n=6 per group), two-way ANOVA (F (1, 12) = 4.879, P = 0.0474), and female (n=6 per group), two-way ANOVA (F (1, 20) = 5.02, P = 0.0365). **(B)** Male daily Food Intake (n=6 per group), Welch’s two-tailed t test (t=2.339, df=9.835, P = 0.0418), and female daily Food Intake (n=6 per group), Welch’s two-tailed t test (t=2735, df=8.417, P = 0.0245). **(C)** Male body composition analysis (n=6 per group), two-tailed unpaired t test (t=2.636, df=7.075, P = 0.033310). **(D)** Male glucose tolerance test and AUC (control n=6, AgRP Fgfr1^Cas9d^ n=6), two-way ANOVA (F (1, 11) = 6.827, P = 0.0241); AUC Welch’s two-tailed t test (t=2.507, df=11, P = 0.0291). **E)** Male insulin tolerance test and AUC (control n=6, AgRP Fgfr1^Cas9d^ n=6), two-way ANOVA (F (1, 11) = 6.522, P = 0.0268); AUC Welch’s two-tailed t test (t=2.640, df=10.72, p= 0.0234). All data are presented as mean ± s.e.m *P < 0.05, **P < 0.01, ***P < 0.001, ****P < 0.0001.

Overexpression of FGFR1 in AgRP neurons reduced weight gain and average daily food intake in both male and female mice when challenged with an HFD (**Figures 2A, B**). Two-way ANOVA revealed significant main effects of AgRP FGFR1 overexpression and sex on body weight and food intake, with no significant sex × AgRP FGFR1 overexpression treatment interactions (**Supplementary Table 2**), indicating that AgRP FGFR1 overexpression effects were consistent between males and females. Overexpression of FGFR1 in AgRP neurons led to maintained lean mass and lower fat mass in HFD mice when compared to the control (**Figure 2C**). Furthermore, mice with overexpression of FGFR1 in AgRP neurons also displayed greater glucose tolerance and control (**Figure 2D**) and insulin sensitivity (**Figure 2E**). Taken together, these findings indicate that overexpression of FGFR1 in AgRP neurons of HFD-fed mice was associated with reduced weight gain and adiposity, as well as improved glucose tolerance and insulin sensitivity.

### Overexpression of FGFR1 in AgRP neurons does not change energy expenditure during HFD feeding

**Figure 3:**
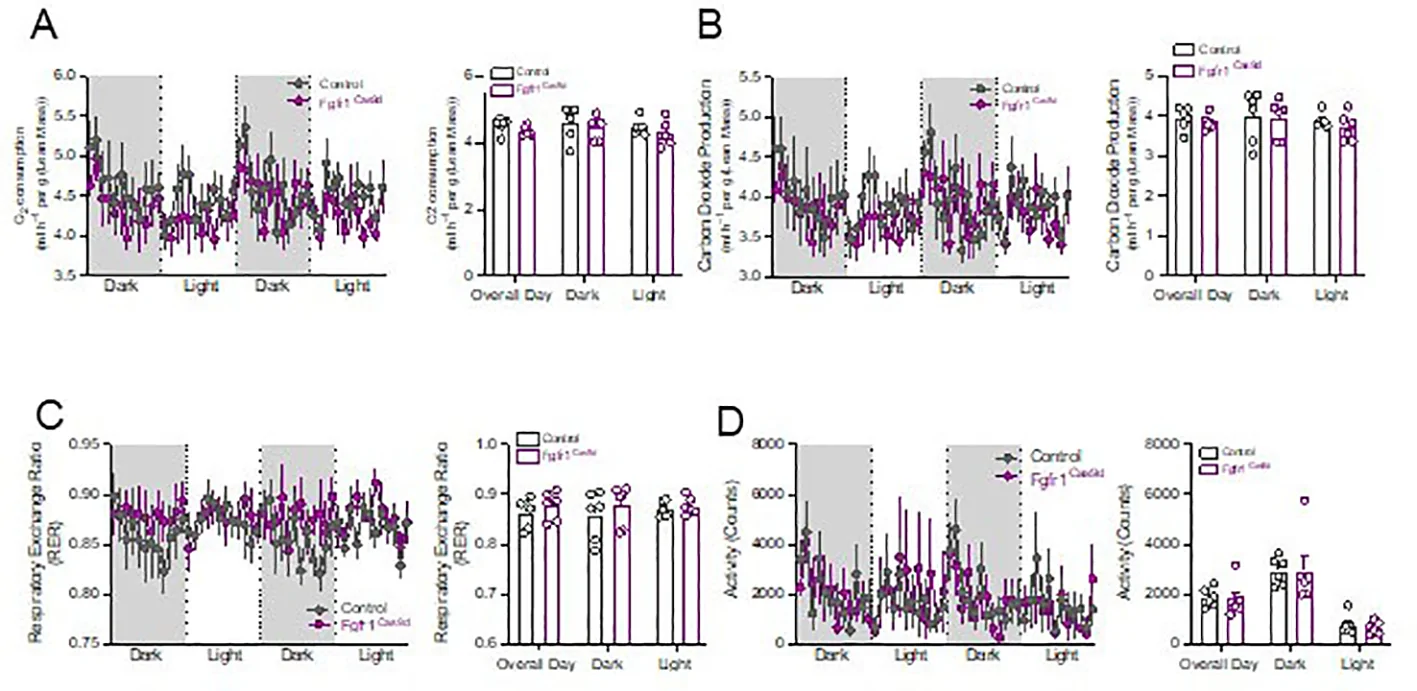
Overexpression of FGFR1 in AgRP neurons does not change energy expenditure during HFD feeding. **(A)** Normalized (Lean Mass) oxygen consumption (n=6 per group), two-way ANOVA (F (1, 10) = 3.473, P = 0.0920). **(B)** Normalized (Lean Mass) carbon dioxide production (n=6 per group), two-way ANOVA (F (1, 10) = 0.7234, P = 0.4149). **(C)** Respiratory exchange ratio (n=6 per group), two-way ANOVA (F (1, 10) = 0.6123, P = 0.4521). **(D)** Locomotion/Activity counts (n=6 per group), two-way ANOVA (F (1, 10) = 0.02262, P = 0.8834). Data are presented as mean ± SEM. *P < 0.05, **P < 0.01.

We next assessed whether FGFR1 overexpression in AgRP neurons affected whole-body metabolism. Comprehensive metabolic phenotyping revealed no significant differences between AgRP neuron FGFR1 overexpression and control HFD-fed mice in oxygen consumption (VO_2_), carbon dioxide production (VCO_2_), or the derived respiratory exchange ratio (RER) across light, dark, and 24-hour cycles (**Figures 3A–C**, **Supplementary Figure 2**). Furthermore, locomotor activity was also comparable between the two groups (**Figure 3D**). Additionally, energy expenditure parameters (VO_2_ and VCO_2_) were analyzed using ANCOVA with lean mass as a covariate, following verification of the homogeneity of regression slopes assumption. RER and locomotor activity were analyzed using unpaired t-tests, as these parameters are not typically adjusted for body composition (**Table 1**, **Supplementary Table 3**). Collectively, these data suggest that overexpression of FGFR1 in AgRP neurons does not alter energy expenditure during HFD feeding.

**Table 1:** Metabolic parameters in control and OE mice.

Parameter	Control (n=6)	OE (n=6)	P value	Method
Lean mass (g)	18.16 ± 0.41	18.81 ± 0.35	0.246	t-test
VO_2_ (mL/h)				
- Light	79.68 ± 4.53	75.38 ± 4.68	0.324	ANCOVA
- Dark	78.96 ± 3.64	83.31 ± 3.73	0.216	ANCOVA
- 24h	77.86 ± 3.27	80.83 ± 3.23	0.221	ANCOVA
VCO_2_ (mL/h)				
- Light	70.03 ± 2.27	72.03 ± 2.27	0.324	ANCOVA
- Dark	71.77 ± 4.59	76.77 ± 4.62	0.531	ANCOVA
- 24h	70.84 ± 2.15	74.37 ± 2.51	0.08	ANCOVA
RER				
- Light	0.8717 ± 0.01	0.8767 ± 0.01	0.63	t-test
- Dark	0.863 ± 0.02	0.875 ± 0.02	0.83	t-test
- 24h	0.868 ± 0.01	0.874 ± 0.01	0.647	t-test
Activity (counts)				
- Light	777.4 ± 164	662.9 ± 101	0.567	t-test
- Dark	2929 ± 226	2947 ± 579	0.978	t-test
- 24h	1869 ± 153	1805 ± 287	0.85	t-test

Data are shown as adjusted means ± SEM after ANCOVA with lean mass as a covariate (VO_2_ and VCO_2_) or as raw means ± SEM (RER and Activity). Lean mass was compared using an unpaired t-test. SEM, standard error of the mean. No significant differences were observed between groups for any parameter (all P > 0.05).

### Overexpression of FGFR1 in AgRP neurons leads to increased FOXO1 phosphorylation and AgRP mRNA downregulation

**Figure 4:**
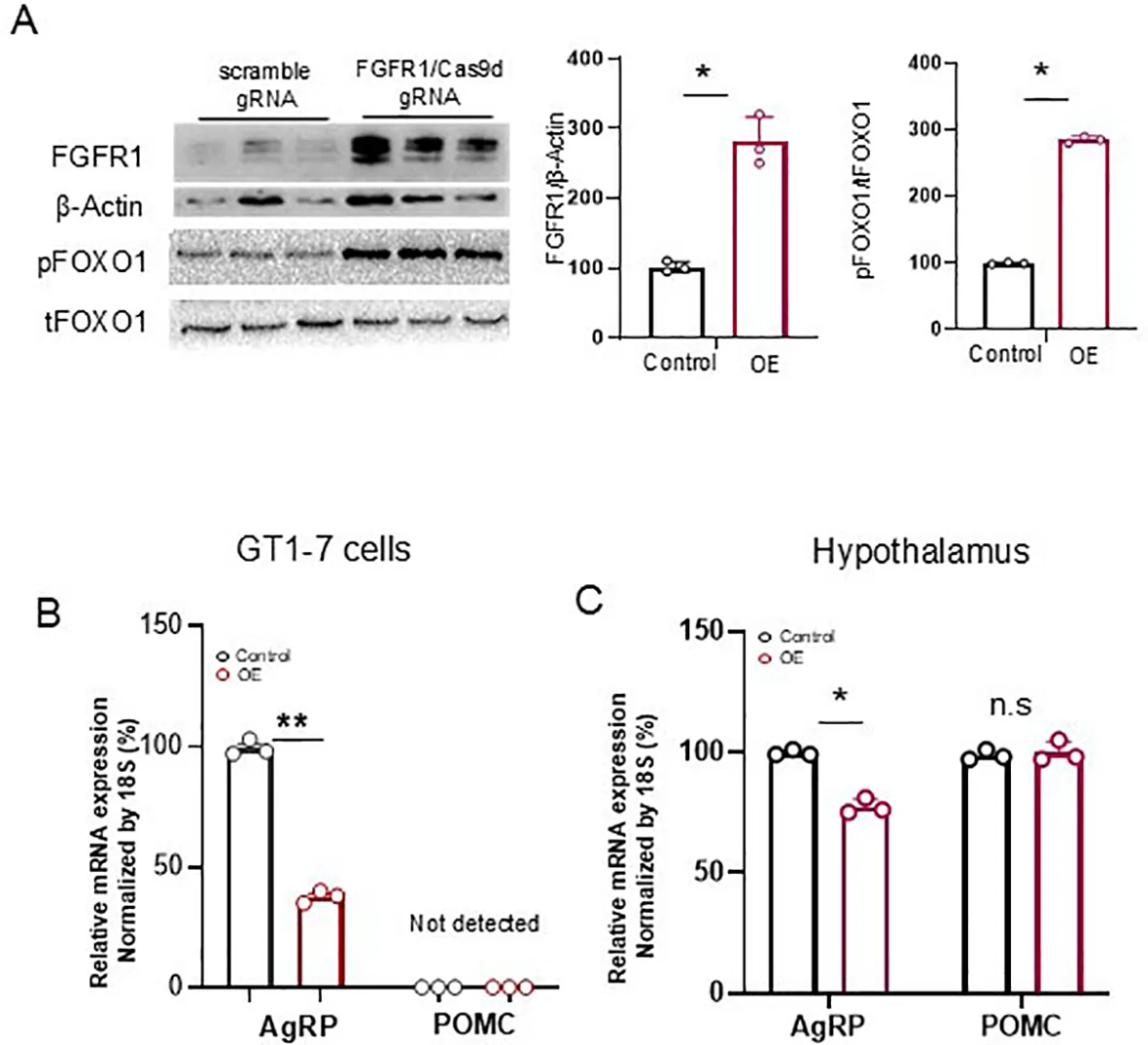
Overexpression of FGFR1 in AgRP neurons leads to increased FOXO1 phosphorylation and AgRP mRNA downregulation. **(A)** Immunoblot showing overexpression of FGFR1 by AAV-Cre/CRISPR/Cas9d increases phosphorylation of FOXO1 in GT1–7 hypothalamic cell lines. Quantification of FGFR1 expression (n=3 per group, two-tailed unpaired t-test t=7.194, df=2, P = 0.0188). Quantification of pFOXO1. (N = 3 per group, two-tailed unpaired t test t=61.51, df=2, P<0.0001). **(B)** AgRP mRNA expression from the GT1–7 overexpression of FGFR1 by AAV-Cre/CRISPR/Cas9d (n=3 per group, two-tailed Welch’s t test t=26.16, df=3.782, P<0.0001). **(C)** AgRP mRNA expression from the whole hypothalamus of AAV-injected mice (n=3 per group, two-tailed Welch’s t test t=18.58, df=2, P = 0.0029). POMC mRNA expression from the whole hypothalamus of AAV-injected mice (n=3 per group, two-tailed Welch’s t test t=0.4500, df=2, P = 0.6968). Data are presented as mean ± s.e.m. *P < 0.05, **P < 0.01.

To elucidate the molecular mechanisms by which FGFR1 signaling in AgRP neurons influences energy balance, we investigated its role in activating the PI3K-FOXO1 pathway, a key regulator of AgRP expression. We first employed CRISPR-Cas9a-mediated overexpression of FGFR1 in hypothalamic GT1–7 cells. In this model, FGFR1 overexpression significantly increased FOXO1 phosphorylation (**Figure 4A**) and decreased AgRP mRNA expression (**Figure 4B**), consistent with the activation of this pathway.

To validate these findings *in vivo*, we analyzed hypothalamic gene expression in mice with an AgRP neuron-specific overexpression of FGFR1. The overexpression of FGFR1 resulted in a significant downregulation of AgRP mRNA (**Figure 4C**). In contrast, the mRNA expression of POMC, a functionally antagonistic neuropeptide, remained unchanged (**Figure 4C**).

Taken together, these *in vitro* and *in vivo* results support a role for FGFR1 signaling in AgRP neurons in energy balance, potentially through the PI3K–FOXO1 axis to regulate AgRP expression. This pathway may thus contribute to the FGFR1-dependent effects on feeding, weight gain, and glycemia.

## Discussion

This study demonstrates that targeted overexpression of FGFR1 specifically in AgRP neurons of the hypothalamus confers significant metabolic benefits, including protection against high-fat diet (HFD)-induced weight gain and adiposity, alongside improved glucose tolerance and insulin sensitivity. Collectively, these findings provide evidence that FGFR1 signaling within this discrete neuronal population may play a role in the regulation of energy and glucose homeostasis.

Our results align with and extend previous work highlighting the importance of FGFR signaling in metabolism. Prior studies have established that FGFR1 is abundantly expressed throughout the hypothalamus (20) and that its ligands can counteract diabetic and obese phenotypes (13, 20). The novel contribution of our study is the precise delineation of FGFR1’s role within AgRP neurons, moving beyond ligand-based approaches. The protective effects we observed are consistent with the interpretation that FGFR1 expression in these neurons may help promote a metabolically favorable state. This is noteworthy given that AgRP neuron activity is potently orexigenic (7, 8); Moreover, we recently reported that lack of FGFR1 in AgRP neurons depolarized resting membrane potential compared with control (22). It is tempting to speculate that FGFR1 signaling could act to counterbalance this drive, possibly by modulating neuronal excitability or neuropeptide release. However, the present data do not directly address the electrophysiological or secretory consequences of FGFR1 overexpression, and further studies are needed to test this hypothesis.

At the molecular level, we observed that FGFR1 signaling is associated with increased FOXO1 phosphorylation in hypothalamic GT1–7 cells, which in turn is known to repress AgRP gene expression (32). This raises the possibility that the FGFR1–FOXO1 axis may represent a key transcriptional pathway through which increased FGFR1 expression could exert a chronic inhibitory tone to suppress AgRP. It is also important to acknowledge that our *in vitro* data are derived exclusively from a single cell line, which may not fully capture the physiological diversity of native neurons. Nevertheless, as we did not directly measure FOXO1 activity or AgRP peptide levels *in vivo*, and because our cell-line observations should be viewed as supportive rather than conclusive evidence, this proposed mechanism remains speculative and requires direct experimental validation *in vivo*.

We also considered the possibility that the hypothalamic–pituitary–adrenal (HPA) axis could be involved, as AgRP neurons are known to activate this axis and serve as a critical mediator of the metabolic response to stress (33). AgRP neurons project to nuclei involved in stress integration, and their activity can influence corticosterone release and stress-coping behaviors. Notably, accumulating evidence indicates that the glucocorticoid receptor (GR) is selectively expressed in AgRP neurons, whereas POMC neurons appear to lack GR expression (34, 35). This distinction suggests that AgRP neurons may function as a key node for direct steroid feedback, potentially allowing them to couple peripheral glucocorticoid levels with hypothalamic output. Concurrently, several FGF ligands have been linked to glucocorticoid regulation and improved glycemic control (13, 36), leading us to hypothesize that FGFR1 activation within AgRP neurons might contribute to attenuation of HPA axis drive under conditions of metabolic stress. If this were the case, such attenuation could occur either through cell-autonomous effects on AgRP excitability or via non-autonomous GABAergic modulation of corticotropin-releasing hormone (CRH) neurons in the paraventricular nucleus of the hypothalamus. Importantly, our study did not directly measure HPA axis function, corticosterone levels, or CRH neuron activity. Thus, while the improvements in glucose homeostasis we observed are consistent with this hypothesis, the present data do not permit any firm conclusions regarding HPA axis involvement. Future studies directly measuring corticosterone levels and stress reactivity in this model, including targeted disruption of GR in AgRP neurons, will be required to determine whether FGFR1 in AgRP neurons influences HPA axis function.

A notable finding was the lack of a statistically significant difference in energy expenditure (as measured by VO_2_, VCO_2_, RER, and activity) between control and experimental groups under HFD conditions. This observation suggests that the anti-obesity effect of AgRP neuron-specific FGFR1 overexpression may be primarily mediated through mechanisms other than a basal increase in whole-body energy expenditure. This result is intriguing, as stimulation of AgRP neurons is known to reduce energy expenditure (7, 8). Given that FGFR1 signaling is thought to inhibit AgRP neuron activity, one might have predicted an increase in energy expenditure. The absence of such an effect could be due to several factors. The metabolic cage measurements were conducted in a resting state without metabolic challenges, such as acute exercise or cold exposure, which might be necessary to reveal differences in energy mobilization capacity. It remains possible that FGFR1 overexpression could enhance metabolic flexibility or the ability to adapt energy expenditure to demand, rather than elevating the baseline rate, although this possibility awaits direct testing.

Several limitations of our study should be considered. First, while the AAV-CRISPRa system allows for targeted adult-onset overexpression, it is possible that the level of FGFR1 expression achieved may not fully recapitulate physiological signaling dynamics. Second, as mentioned, the assessment of energy expenditure was performed under standard housing conditions; exposing these mice to metabolic stressors could provide a more comprehensive picture. Finally, while our findings suggest that AgRP neuron-specific FGFR1 overexpression improves diabetic parameters, a critical question remains: are these metabolic effects primarily driven by direct FGFR1 signaling within AgRP neurons, or are they largely secondary to the observed reductions in food intake and/or adiposity? Distinguishing direct, neuron-autonomous mechanisms from indirect, adiposity-dependent effects will be an important objective for future research.

In conclusion, our findings provide evidence that enhancing FGFR1 signaling specifically within AgRP neurons can ameliorate diet-induced metabolic dysfunction in this experimental model. This work shifts the focus from exogenous ligands to the receptor itself, suggesting that FGFR1 in AgRP neurons may represent a potential nodal point for therapeutic intervention in metabolic disorders, including type 2 diabetes and obesity. However, as discussed above, the mechanistic foundation of these effects—particularly regarding FOXO1 phosphorylation, AgRP regulation, and HPA axis involvement—remain largely correlative and require direct experimental confirmation. Future investigations should aim to delineate the precise molecular and circuit mechanisms by which FGFR1 signaling in AgRP neurons influences systemic metabolism, including its interaction with the HPA axis. Furthermore, exploring how endogenous FGFR1 ligands function within this specific neuronal context will be crucial for determining whether these findings can be translated into novel therapeutic approaches.

## Data Availability

The original contributions presented in the study are included in the article/**Supplementary Material**. Further inquiries can be directed to the corresponding author.

## Supplementary material

Supplementary Figure 1 Viral Strategy and validation of CRISPR/Cas9a-mediated overexpression of FGFR1 in AgRP neurons. After fluorescence-activated cell sorting (FACS), **(A)** FGFR1, 2, and 3 mRNA expression following CRISPR-Cas9a-mediated overexpression of FGFR1 in non-AgRP (mCherry negative) neurons, n=3 per group; no significance was observed. **(B)** AgRP mRNA expression in mCherry-Positive and Negative neurons following CRISPR-Cas9a-mediated overexpression of FGFR1, n=3 per group. ND: Not detected.

Supplementary Figure 2 Overexpression of FGFR1 in AgRP neurons does not change energy expenditure during HFD feeding. **(A)** Raw oxygen consumption (n=6 per group), ANOVA (F (1, 432) = 0.3696, P = 0.5435). **(B)** Raw carbon dioxide production (n=6 per group), two-way ANOVA (F (1, 480) = 0.03389, P = 0.8540) in control and AgRP neurons FGFR1 overexpression mice. Data are presented as mean ± SEM. **P* < 0.05, ***P* < 0.01.

Supplementary Table 1 Primer Sequences used in Quantitative real-time PCR.

Supplementary Table 2 Body weight and food intake in male and female Control and OE (AgRP neuron FGFR1 overexpression) mice. Data are shown as mean ± SD (n = 6 per group for each sex and AgRP neuron FGFR1 overexpression combination). Two-way ANOVA was performed with sex and AgRP neuron FGFR1 overexpression as factors. P values for main effects and interaction are shown. No significant sex × AgRP neuron FGFR1 overexpression interactions were observed (body weight: P = 0.895; food intake: P = 0.91), indicating that treatment (AgRP neuron FGFR1 overexpression) effects were consistent between males and females.

Supplementary Table 3 Individual CalR parameters for all animals. All values represent raw data from individual animals. VO_2_ = oxygen consumption; VCO_2_ = carbon dioxide production; RER = respiratory exchange ratio; Activity = locomotor activity counts.

## References

[1] Zhang Y Proenca R Maffei M Barone M Leopold L Friedman JM . Positional cloning of the mouse obese gene and its human homologue. Nature. (1994) 372:425–32. doi: 10.1038/372425a0 7984236

[2] Kojima M Kangawa K . Ghrelin: structure and function. Physiol Rev. (2005) 85:495–522. doi: 10.1152/physrev.00012.2004 15788704

[3] Andermann ML Lowell BB . Toward a wiring diagram understanding of appetite control. Neuron. (2017) 95:757–78. doi: 10.1016/j.neuron.2017.06.014 28817798 PMC5657399

[4] Kim JD Leyva S Diano S . Hormonal regulation of the hypothalamic melanocortin system. Front Physiol. (2014) 5:480. doi: 10.3389/fphys.2014.00480 25538630 PMC4260486

[5] Morton GJ Cummings DE Baskin DG Barsh GS Schwartz MW . Central nervous system control of food intake and body weight. Nature. (2006) 443:289–95. doi: 10.1038/nature05026 16988703

[6] Landry T Laing BT Li P Bunner W Rao Z Prete A . Central α-Klotho suppresses NPY/AgRP neuron activity and regulates metabolism in mice. Diabetes. (2020) 69:1368–81. doi: 10.2337/db19-0941 32332158 PMC7306125

[7] Aponte Y Atasoy D Sternson SM . AGRP neurons are sufficient to orchestrate feeding behavior rapidly and without training. Nat Neurosci. (2011) 14:351–5. doi: 10.1038/nn.2739 21209617 PMC3049940

[8] Krashes MJ Koda S Ye C Rogan SC Adams AC Cusher DS . Rapid, reversible activation of AgRP neurons drives feeding behavior in mice. J Clin Invest. (2011) 121:1424. doi: 10.1172/jci46229 21364278 PMC3069789

[9] Tan B Hedbacker K Kelly L Zhang Z Moura-Assis A Luo JD . A cellular and molecular basis of leptin resistance. Cell Metab. (2025) 37:723–741.e6. doi: 10.1016/j.cmet.2025.01.001 40043692

[10] Lee SH Park SY Choi CS . Insulin resistance: from mechanisms to therapeutic strategies. Diabetes Metab J. (2022) 46:15–37. doi: 10.4093/dmj.2021.0280 34965646 PMC8831809

[11] Reuss B von Bohlen und Halbach O . Fibroblast growth factors and their receptors in the central nervous system. Cell Tissue Res. (2003) 313:139–57. doi: 10.1007/s00441-003-0756-7 12845521

[12] Gonzalez AM Berry M Maher PA Logan A Baird A . A comprehensive analysis of the distribution of FGF-2 and FGFR1 in the rat brain. Brain Res. (1995) 701:201–26. doi: 10.1016/0006-8993(95)01002-x 8925285

[13] Scarlett JM Rojas JM Matsen ME Kaiyala KJ Stefanovski D Bergman RN . Central injection of fibroblast growth factor 1 induces sustained remission of diabetic hyperglycemia in rodents. Nat Med. (2016) 22:800–6. doi: 10.1038/nm.4101 27213816 PMC4938755

[14] Ryan KK Kohli R Gutierrez-Aguilar R Gaitonde SG Woods SC Seeley RJ . Fibroblast growth factor-19 action in the brain reduces food intake and body weight and improves glucose tolerance in male rats. Endocrinology. (2013) 154:9–15. doi: 10.1210/en.2012-1891 23183168 PMC3529386

[15] Marcelin G Jo YH Li X Schwartz GJ Zhang Y Dun NJ . Central action of FGF19 reduces hypothalamic AGRP/NPY neuron activity and improves glucose metabolism. Mol Metab. (2014) 3:19–28. doi: 10.1016/j.molmet.2013.10.002 24567901 PMC3929918

[16] Sarruf DA Thaler JP Morton GJ German J Fischer JD Ogimoto K . Fibroblast growth factor 21 action in the brain increases energy expenditure and insulin sensitivity in obese rats. Diabetes. (2010) 59:1817–24. doi: 10.2337/db09-1878 20357365 PMC2889784

[17] Jensen-Cody SO Flippo KH Claflin KE Yavuz Y Sapouckey SA Walters GC . FGF21 signals to glutamatergic neurons in the ventromedial hypothalamus to suppress carbohydrate intake. Cell Metab. (2020) 32:273–286.e6. doi: 10.1016/j.cmet.2020.06.008 32640184 PMC7734879

[18] Flippo KH Potthoff MJ . Metabolic messengers: FGF21. Nat Metab. (2021) 3:309–17. doi: 10.1038/s42255-021-00354-2 33758421 PMC8620721

[19] Brown JM Scarlett JM Matsen ME Nguyen HT Secher A Jorgensen R . The hypothalamic arcuate nucleus–median eminence is a target for sustained diabetes remission induced by fibroblast growth factor 1. Diabetes. (2019) 68:1054–61. doi: 10.2337/db19-0025 30796029 PMC6477902

[20] Sun H Lin W Tang Y Tu H Chen T Zhou J . Sustained remission of type 2 diabetes in rodents by centrally administered fibroblast growth factor 4. Cell Metab. (2023) 35:1022–1037.e6. doi: 10.1016/j.cmet.2023.04.018 37167965

[21] Shookster D Landry T Bunner W O’Connell S Paterl D Huang H . Deletion of FGFR1 in hypothalamic neurons alters energy homeostasis and negates the metabolic effects of α-Klotho. Endocrinology. (2026) 167:bqaf182. doi: 10.1210/endocr/bqaf182 41408652 PMC12755302

[22] Shookster D O’Connell S Patel D Landry T Bunner W Jiang Z . Selective deletion of FGFR1 in AgRP neurons impairs energy and glucose homeostasis under high-fat diet in mice. Mol Metab. (2026) 105:102332. doi: 10.1016/j.molmet.2026.102332 41679434 PMC12936525

[23] Konermann S Brigham MD Trevino AE Joung J Abudayyeh OO Barcena C . Genome-scale transcriptional activation by an engineered CRISPR-Cas9 complex. Nature. (2015) 517:583–8. doi: 10.1038/nature14136 25494202 PMC4420636

[24] Dominguez AA Lim WA Qi LS . Beyond editing: repurposing CRISPR-Cas9 for precision genome regulation and interrogation. Nat Rev Mol Cell Biol. (2015) 17:5–15. doi: 10.1038/nrm.2015.226 26670017 PMC4922510

[25] Di Maria V Moindrot M Ryde M Bono A Quintino L Ledri M . Development and validation of CRISPR activator systems for overexpression of CB1 receptors in neurons. Front Mol Neurosci. (2020) 13:168. doi: 10.3389/fnmol.2020.00168 33013319 PMC7506083

[26] Müller TD Klingenspor M Tschöp MH . Revisiting energy expenditure: how to correct mouse metabolic rate for body mass. Nat Metab. (2021) 3:1134–6. doi: 10.1038/s42255-021-00451-2 34489606

[27] Mina AI LeClair RA LeClair KB Cohen DE Lantier L Banks AS . CalR: a web-based analysis tool for indirect calorimetry experiments. Cell Metab. (2018) 28:656–661.e1. doi: 10.2139/ssrn.3155918 30017358 PMC6170709

[28] Ratz M von Berlin L Larsson L Martin M Westholm JO La Manno G . Clonal relations in the mouse brain revealed by single-cell and spatial transcriptomics. Nat Neurosci. (2022) 25:285–94. doi: 10.1038/s41593-022-01011-x 35210624 PMC8904259

[29] Brewer JR Molotkov A Mazot P Hoch RV Soriano P . Fgfr1 regulates development through the combinatorial use of signaling proteins. Genes Dev. (2015) 29:1863–74. doi: 10.1101/gad.264994.115 26341559 PMC4573858

[30] Kalamakis G Platt RJ . CRISPR for neuroscientists. Neuron. (2023) 111:2282–311. doi: 10.1016/j.neuron.2023.04.021 37201524

[31] Xu J Bartolome CL Low CS Yi X Chien CH Wang P . Genetic identification of leptin neural circuits in energy and glucose homeostasis. Nature. (2018) 556:505–9. doi: 10.1038/s41586-018-0049-7 29670283 PMC5920723

[32] Kitamura T Feng Y Kitamura YI Chua SC Jr Jr. Xu AW . Forkhead protein FoxO1 mediates Agrp-dependent effects of leptin on food intake. Nat Med. (2006) 12:534–40. doi: 10.1038/nm1392 16604086

[33] Douglass AM Resch JM Madara JC Kucukdereli H Yizhar O Grama A . Neural basis for fasting activation of the hypothalamic-pituitary-adrenal axis. Nature. (2023) 620:154–62. doi: 10.1038/s41586-023-06358-0 37495689 PMC11168300

[34] Yesmin R Watanabe M Sinha AS Ishibashi M Wang T Fukuda A . A subpopulation of agouti-related peptide neurons exciting corticotropin-releasing hormone axon terminals in median eminence led to hypothalamic-pituitary-adrenal axis activation in response to food restriction. Front Mol Neurosci. (2022) 15:990803. doi: 10.3389/fnmol.2022.990803 36245920 PMC9557964

[35] Leon-Mercado L Herrera Moro Chao D Basualdo MD Kawata M Escobar C Buijs RM . The arcuate nucleus: a site of fast negative feedback for corticosterone secretion in male rats. eNeuro. (2017) 4:ENEURO.0350–16.2017. doi: 10.1523/ENEURO.0350-16.2017 28275717 PMC5334455

[36] Perry RJ Lee S Ma L Zhang D Schlessinger J Shulman GI . FGF1 and FGF19 reverse diabetes by suppression of the hypothalamic–pituitary–adrenal axis. Nat Commun. (2015) 6:6980. doi: 10.1038/ncomms7980 25916467 PMC4413509

